# Application of IVIM in the assessment of the spleen in patients with myelofibrosis

**DOI:** 10.3389/fonc.2025.1652543

**Published:** 2026-01-14

**Authors:** Mateusz Bilski, Marta Sobas, Aleksander Pawluś, Marek Skarupski, Anna Zimny

**Affiliations:** 1 Department of Radiology, Wroclaw Medical University, Wrocław, Poland; 2 Department of Hematology, Collegium Medicum in Bydgoszcz, Nicolaus Copernicus University in Toruń, Bydgoszcz, Poland; 3 Specialist Hospital in Legnica, Legnica, Poland; 4 Faculty of Pure and Applied Mathematics, Wrocław University of Science and Technology, Wrocław, Poland; 5 Department of Mathematics and Computer Science, Eindhoven University of Technology, Eindhoven, Netherlands

**Keywords:** IVIM, myelofibrosis, myeloproliferative neoplasms, non-invasive diagnostics, spleen, spleen volume

## Abstract

**Aim:**

The purpose of this study is to investigate whether IVIM-derived spleen parameters including the true diffusion coefficient (D) and perfusion fraction (f) can serve as non-invasive biomarkers to predict fibrotic transformation of the bone marrow.

**Methods:**

Eighteen patients with MPN and 18 healthy controls (HC) underwent abdominal IVIM diffusion-weighted imaging using 1.5 T MR unit. The values of spleen parameters of D and f were compared between patients with various forms of MPN, including 8 patients with myelofibrosis (MF), 10 patients without myelofibrosis (non-MF) and (HC). A Bayesian approach was adopted in the analysis, to assess whether the D and f coefficients, along with the spleen volume, can predict disease occurrence.

**Results:**

Comparing MF patients to HC, Bayesian analysis provided strong evidence for a difference in the f parameter (BF = 18.38), while the difference between MF and non-MF patients was weaker (BF = 1.42). There was no statistically significant difference between non-MF and HC (BF = 0. 64). There was no correlation between f parameter and spleen volume (CI= -0,43; 0,38). Logistic regression analysis with logkit function confirmed that f parameter was significant predictor of MF.

**Conclusions:**

The study demonstrates that IVIM measurements of spleen perfusion in both healthy individuals and MPN patients offer valuable predictive information regarding disease development and progression. The f parameter shows promise for predicting fibrotic transformation of the bone marrow in MPN patients regardless of the size of the spleen.

## 1 Introduction

Myelofibrosis is a progressive bone marrow disorder marked by fibrotic transformation, extramedullary hematopoiesis, cytopenias, and constitutional symptoms (1, 2). It predominantly arises in the context of myeloproliferative neoplasms, including primary myelofibrosis (PM), post-essential thrombocythemia myelofibrosis (PETM), and post-polycythemia vera myelofibrosis (PPVM), and it may also appear in other advanced myeloid malignancies (2, 3). These diseases often harbor certain mutations causing progressive marrow fibrosis, clonal expansion of hematopoietic stem cells, and deregulated cytokine production (1–3). Myelofibrosis assessment involves histopathological examination of the bone marrow, as well as genetic analysis, and application of prognostic tools such as GIPSS or MIPSS70. Imaging techniques are frequently used to evaluate extramedullary disease (2–4).

Intravoxel incoherent motion (IVIM) is an advanced MRI method that separates the random motion of water molecules from microcirculatory blood flow by acquiring multiple diffusion-weighted images at different gradient strengths, thereby allowing one to capture both “true” diffusion and perfusion components within tissues (5, 6). Mathematical modeling of the so called signal decay curve, allows calculations of the IVIM parameters, including pure diffusion coefficient (D), sensitive to tissue cellularity, the pseudo-diffusion coefficient (D*), reflecting capillary blood flow velocity, and the perfusion fraction (f), that indicate the proportion of vascular flow that contributes to the overall signal (7–9). The IVIM parameters can be extracted non-invasively, especially without the use of contrast agents, being a valuable tool for assessing various organs and pathologies in a robust and reproducible manner (5–10).

Assessing MF presents considerable challenges due to overlapping features with other MPNs, the need for invasive bone marrow biopsies, and the complex transition from early stages to secondary overt disease (1–4). The diagnostic difficulties make it important to search for sensitive and non-invasive methods that could refine diagnostic accuracy, improve prognostic predictions, and improve prognostic predictions (11–16). Histopathological examination of bone marrow remains the diagnostic gold standard in MF. Non-invasive methods could refine diagnostic accuracy and support clinical decision-making. This includes assessment of megakaryocytic proliferation and atypia, with or without reticulin fibrosis, as well as granulocytic hyperplasia and decreased erythropoiesis. Imaging techniques, especially IVIM may offer complementary information to biopsy results, particularly they can be used to clarify disease stage, and guide individualized treatment strategies (2, 3, 17–20).

Previous studies identified spleen volume as a promising predictor of MF progression, especially when splenomegaly develops in both primary and secondary MF (21–24). In addition, investigations of the bone marrow–liver–spleen axis reinforce the importance of spleen condition for understanding MPNs pathophysiology and disease evolution (4, 20, 25–30). Various research efforts also highlight that spleen alterations may correlate to high extent with overall morbidity and survivability in MPNs (31–33).

Several investigations explored IVIM-derived parameters in the spleen, as a promising non-invasive technique, not employing contrast agents. They primarily focused on perfusion-related indices and their correlation with severity of the pathological process (4, 9, 10, 25, 26, 31–34). IVIM demonstrated good reproducibility in evaluating tissue microcirculation in the spleen and other organs so far, including in myeloid malignancies (5, 35–39). Preliminary data suggested that IVIM-based spleen imaging might become an innovative diagnostic or prognostic tool, complementing the established use of spleen volume measurements in MF and other MPNs (6–8, 40–42).

Despite the above findings, based on our current knowledge, no research comprehensively examined whether spleen-based IVIM measurements can serve as early predictors of bone marrow fibrotic transformation in MPN patients who had not yet developed overt MF, creating an obvious gap in our understanding of subclinical disease progression and the usefulness of the IVIM parameters as biomarkers that can serve as disease predictor and fibrotic transformation.

This original study aims to investigate whether IVIM-derived spleen parameters can predict fibrotic transformation in the bone marrow of both healthy individuals and MPN patients without MF. Bone marrow blood flow measurements using the IVIM technique (including the true diffusion coefficient (D) and perfusion fraction (f)) will be compared between MF patients and other participants (healthy controls and MPN patients without MF) for comparative analysis.

## 2 Materials and methods

### 2.1 Study participants

The study included 36 participants, 18 patients with MPNs and 18 healthy controls (HCs), with the mean age of MPN patients of 55.1 years (range: 26–78), and the mean age of HCs of 59.3 years (range: 34–81). Among healthy individuals, the mean age was 59.3 years (range: 34–81).

The MPN patient group consisted of individuals diagnosed with PV, ET, essential PMF, as well as post-PV or post-ET myelofibrosis. To assess IVIM parameters among patients with different disease stages, recruitment ensured that approximately half of the MPN group had MF. That is why the MPNs group, patients were categorized based on the presence or absence of MF. 8 participants were diagnosed with MF (mean age: 65.5 years, range: 53–78) and other 10 were non-MF MPN patients (mean age: 54.8 years, range: 26–81). Among MF patients, 2 individuals with PMF, 5 with post-PV MF, and 1 with post-ET MF were included. The non-MF MPN subgroup consisted of 10 patients with either PV or ET (**Table 1**).

**Table 1:** Presentation of the group of participants.

Characteristic	All MPN patients	MF patients	Non-MF subjects	HC
N	18	8	28	18
Age (mean, range)	55.11 (26-78)	65.5 (53-78)	54.8 (26-81)	59.28 (34-81)
Sex (M/F)	7/11	5/3	10/18	8/10
PMF	2	2	–	–
post PVMF	5	5	–	–
post ET MF	1	1	–	–
Other MPNs (ET, PV)	10	–	10	

MPN, myeloproliferative neoplasms; MF, myelofibrosis; PMF, primary myelofibrosis; HC, healthy controls; post-PV MF, post-polycythemia vera myelofibrosis; post-ET MF, post-essential thrombocythemia myelofibrosis; ET, essential thrombocythemia; PV, polycythemia vera; M, male; F, female.

We underline that PV and ET represent distinct myeloproliferative phenotypes, therefore the groups includes cases with differences in bone marrow morphology, cytokine milieu, and potential splenic hemodynamics. However, due to the limited number of cases in each group, separate subgroup analyses were not supportive. All the diagnoses in this patients were rendered on bone marrow biopsy material, classified according to WHO criteria.

Inclusion criteria for MPN patients required a confirmed diagnosis based on bone marrow biopsy and, in the case of non-MF MPN patients, a lack of bone marrow fibrosis at the time of the study. HCs were included if they had no history of chronic spleen or liver diseases or disorders, prior splenic surgery, or systemic diseases affecting spleen function (e.g., chronic liver disease, cirrhosis, immune system disorders). Exclusion criteria for both groups included age under 18 years and poor-quality MRI images that rendered IVIM measurements unreliable.

Selected clinical variables with potential relevance to splenic perfusion were collected where available and coherently reported in clinical documentation. Among the 17 MPN patients included, 11 were JAK2-positive, 1 was MPL-positive, 1 was CALR-positive, and 3 patients were triple-negative. Cytoreductive therapy was reported in 9 patients at the time of imaging (primarily hydroxyurea). However, clinical information on symptom burden, disease duration, and fibrosis grading was inconsistently available and thus not included in statistical modeling.

Recruitment was conducted between July and October 2023, with MRI acquisitions performed from January 2024 to August 2024. All participants provided written informed consent, as well as were informed of their rights, including the right to withdraw at any time. The study was conducted in accordance with the principles of the Declaration of Helsinki and received approval from the Bioethical Commission (Reference No. KB-21/2024).

### 2.2 Imaging procedure

All participants underwent physical and subjective examination, including standard laboratory tests [Complete blood count (CBC), peripheral blood smear, lactate dehydrogenase (LDH), C-reactive protein (CRP), ferritin, erythrocyte sedimentation rate (ESR), fibrinogen, liver function tests (ALT, AST, ALP, GGT, bilirubin), kidney function tests (creatinine, urea), coagulation profile (PT, APTT, INR), serum iron, total iron-binding capacity (TIBC), transferrin saturation, vitamin B12, folate, reticulocyte count], as well as an imaging evaluation by MRI. In addition, those with MPN had a bone marrow biopsy to confirm or exclude MF according to WHO criteria. The inclusion criteria for all participants required age ≥18 and acceptable image quality, while exclusion criteria comprised age <18 and poor-quality data.

MRI examinations were performed in the supine position using a 1.5 T scanner (Siemens Avanto) with a abdomen coil, starting with any necessary structural sequences. IVIM Diffusion-Weighted Imaging (DWI) was performed via a single-shot spin echo-planar imaging with spectral spatial excitation for fat suppression. The b-values were 0, 10, 20, 40, 60, 100, 200, and 800 s/mm², Repetition was 7800 ms, echo time was 62ms, slice thickness was 5.0 mm, field of view was 380mm, and each scan took about 7 min, depending on breathing.

To minimize respiratory variability, all sequences are free-breathing motion corrected. In all cases of uncertainty, the sequences were repeated.

### 2.3 Imaging analysis

**Figure 1:**
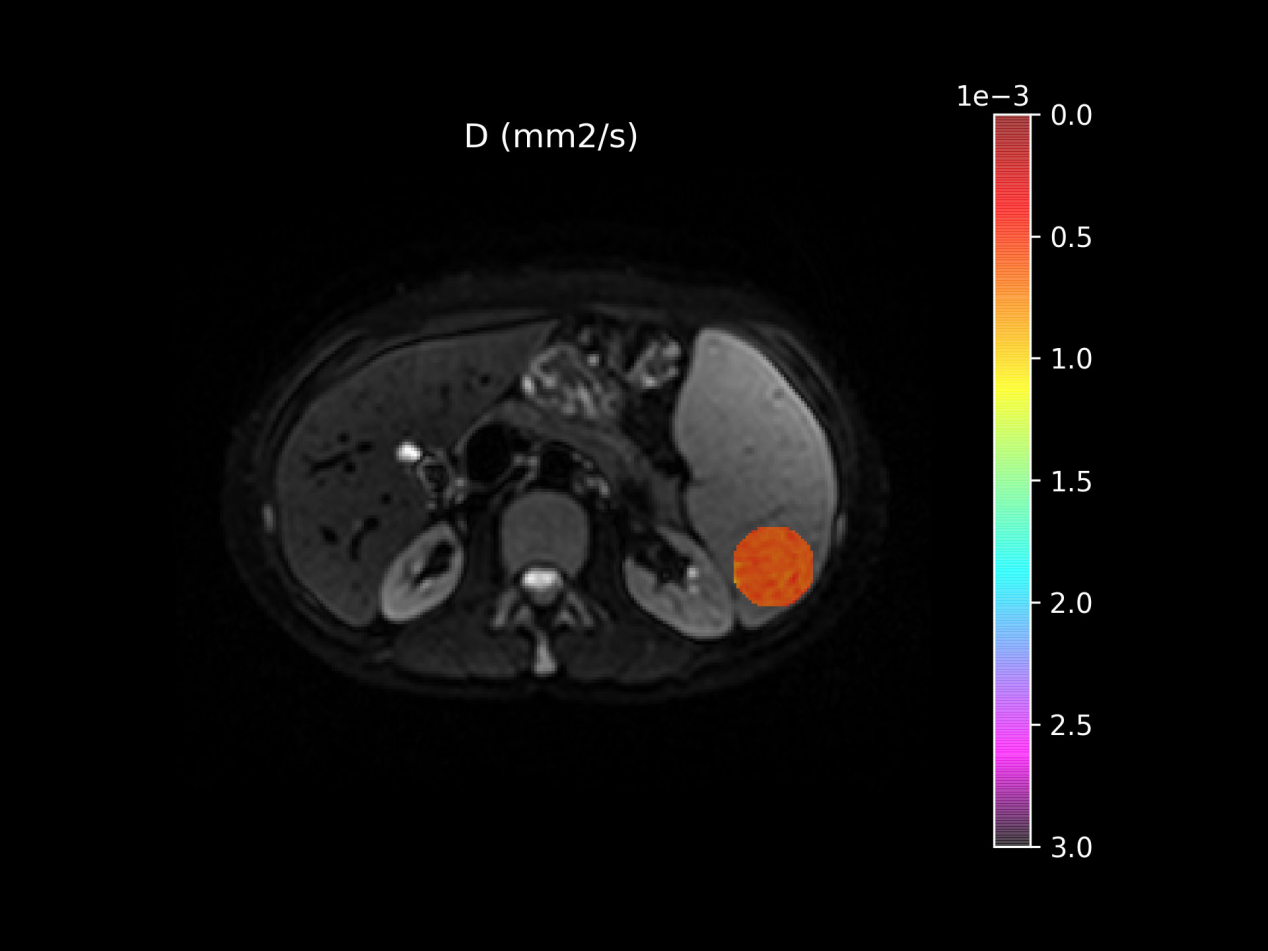
Region of interest placing procedure.

All IVIM images were inspected for artifacts, and those deemed unsatisfactory were excluded. The remaining images were processed using Quibim’s proprietary DWI IVIM algorithm, generating parametric maps of D, f, D*. A region of interest (ROI) was placed manually (**Figure 1** within the spleen parenchyma], avoiding large vessels, the splenic hilum, and artifacts. A region of interest (ROI) was manually placed in the central axial slice of the spleen, using the largest possible circular area that fully encompassed the parenchyma without including vessels, surrounding structures, and artifacts (**Figure 1**) as per technical guidance (consulted and confirmed by the producer of the software). Reproducibility metrics for ROI placement were not included because in the current software version this has not been possible yet. Automated segmentation was not used, however the ROI selections were independently reviewed and confirmed by two additional authors, before the final analysis, to ensure consistency and mitigate operator bias.

No additional motion correction or image registration was applied during post-processing, in accordance with standard manufacturer protocols. No respiratory artifacts were observed.

The spleen volume was calculated using the standard prolate ellipsoid formula (length x thickness x width x0.523).

### 2.4 Statistical analysis

A generalized linear model with a logit link (logistic regression) was used to estimate the probability of myelofibrosis (MF) in relation to IVIM-derived spleen parameters (D and f), assessed both individually and in combination. Model comparisons were based on the Akaike information criterion (AIC), and discriminative ability was assessed using receiver operating characteristic (ROC) curves. A linear regression model with a log link was used to examine the association between spleen volume and IVIM parameters. Model fit and assumptions were assessed using residual plots to check for fitness and normality of errors. The significance level was set at α = 0.05. All statistical analyses were performed in R (version 4.3.3) using the stats, pROC, ROCR, graphics, and ggplot2 packages.

Due to the relatively small sample size, a Bayesian approach was used in key parts of this analysis, involving Markov Chain Monte Carlo (MCMC) sampling with 4000 warm-up and 4000 post-warm-up iterations. Non-informative priors were assumed for the regression coefficients, and convergence was assessed by inspection of residuals and credible intervals. Group comparisons were interpreted based on Bayes factors (BF), using predefined thresholds: BF = 1–3 (weak evidence), BF = 3–10 (moderate), BF = 10–30 (strong), and BF > 30 (very strong evidence).

## 3 Results

Patients with MF have larger spleen measurements compared to those without MF (**Table 1**), whereas f values were notably lower in MF cases. Despite a wide age range in the HC group, D showed no discernible differences among any groups, suggesting f may be a more sensitive marker than D.

When focusing on pairwise comparisons of f-values (**Table 2**), the overall BF (BF = 18.38) for the f parameter between MF and HCs indicated strong evidence for group differences. We further assessed the stability and precision of these estimates given the small MF sample (n = 8). Posterior analysis showed that the estimated difference in f between MF and HC was 0.041 (95% credible interval: [0.012; 0.070]), whereas for MF versus non-MF MPN patients the difference was 0.022 (95% CI: [–0.006; 0.053]). The first comparison excluded zero and yielded a narrow BF range (18.4–20.6), confirming an exceptionally robust evidence. The second comparison exhibited weaker support with a BF range of 1.15–1.42 and a CI that included zero. These indicate that although directional trends were consistent, the strength of inference—particularly for MF vs non-MF—was sensitive to prior specification and sample limitations. In line with this, the estimate for the HC vs non-MF comparison was 0.014 (95% CI: [–0.012; 0.041], BF = 0.64), indicating an overlap between these two groups. Taken together, these findings demonstrate that the observed association between lower f values and MF status is stable for comparisons with HCs, while conclusions for intra-MPN differences should be regarded as preliminary.

**Table 2:** Descriptive statistics of age, and IVIM-derived parameters (D, f) across MF, non-MF subgroups, and the HCs.

Parameter	MF patients	Non-FM subjects	HC	BF
D – value (˙ 10^-4^) Mean (range)	6.182 (5.230; 8.650)	7.195 (5.610; 10.800)	7.102 (5.780; 8.380)	0.97
f – value (˙ 10^-2^) Mean (range)	5.87 (3.00; 9.00)	8.90 (3.00; 18.00)	10.72 (5.00; 18.00)	6.17
Spleen length [cm] Mean (range)	17.93 (12.50; 24.00)	10.81 (7.70; 13.00)	9.89 (6.80; 13.60)	>1000
Spleen volume [cm^3^] Mean (range)	908.6 (364.0; 1283.0)	264.2 (84.0; 456.0)	175.2 (69.0; 335.0)	>1000

MF, myelofibrosis; Non-MF, non-myelofibrosis MPN patients; HC, healthy controls; BF, Bayesian Factor; M, male; F, female.

Multinomial regression analysis (**Table 3**) reinforced the negative correlation between f and MF. The magnitude of the effect was more substantial for MF (Y = 2) than for the non-MF subgroup (Y = 1). The model’s credible intervals excluded zero, indicating robust statistical support for finding f as a predictor of MF.

**Table 3:** Pairwise Bayes Factors for f parameter comparisons, including the subgroups.

Parameter	Comparison	BF	BF range	Effect size (Mean diff.)	95% CI
F	HCs vs. Non-MF	0.640	[0.416 – 0.640]	0.014	[–0.012; 0.041]
F	HCs vs. MF	18.379	[18.379 – 20.632]	0.041	[0.012; 0.070]
F	Non-MF vs. MF	1.420	[1.150 – 1.420]	0.022	[–0.006; 0.053]

HC, healthy controls; Non-MF, non-myelofibrosis MPN patients; MF, myelofibrosis.

In examining how f and spleen volume vary together, modeling spleen volume as a function of f revealed relatively stronger explanatory power than inverse relationship (**Table 4**).

**Table 4:** Multinomial logistic regression estimates for f parameter, including the subgroups.

Parameter	Estimate	95% CI	Est. Error	$\hat{R}$
µ_1_	-7.76	-16.39; -0.13	4.21	1
µ_2_	-14.87	-26.84; -4.83	5.58	1

**Figure 2:**
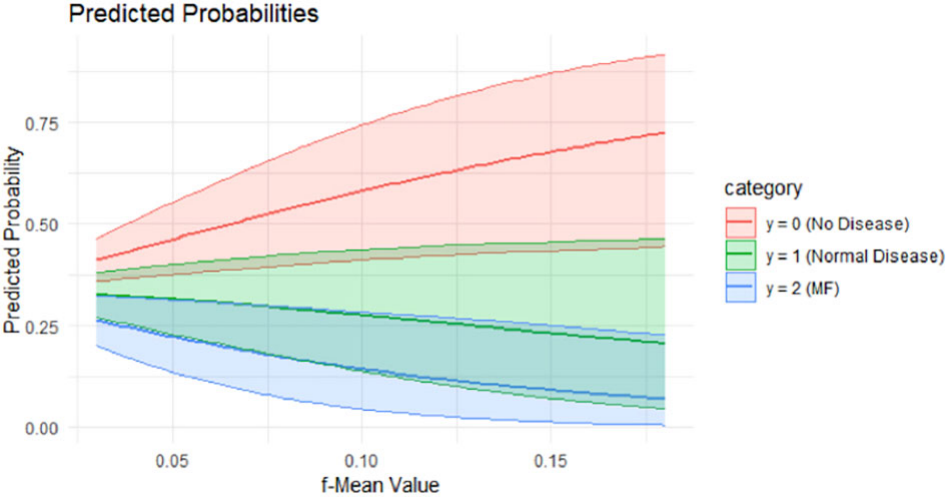
Predicted probabilities of f-Mean value across the study subgroups and HCs.

**Figure 2** shows the predicted probabilities of MF status in relation to the mean f values across the subgroups. The plot illustrates that lower f values are strongly associated with a higher probability of MF, highlighting f (potential discriminative marker).

**Figure 3:**
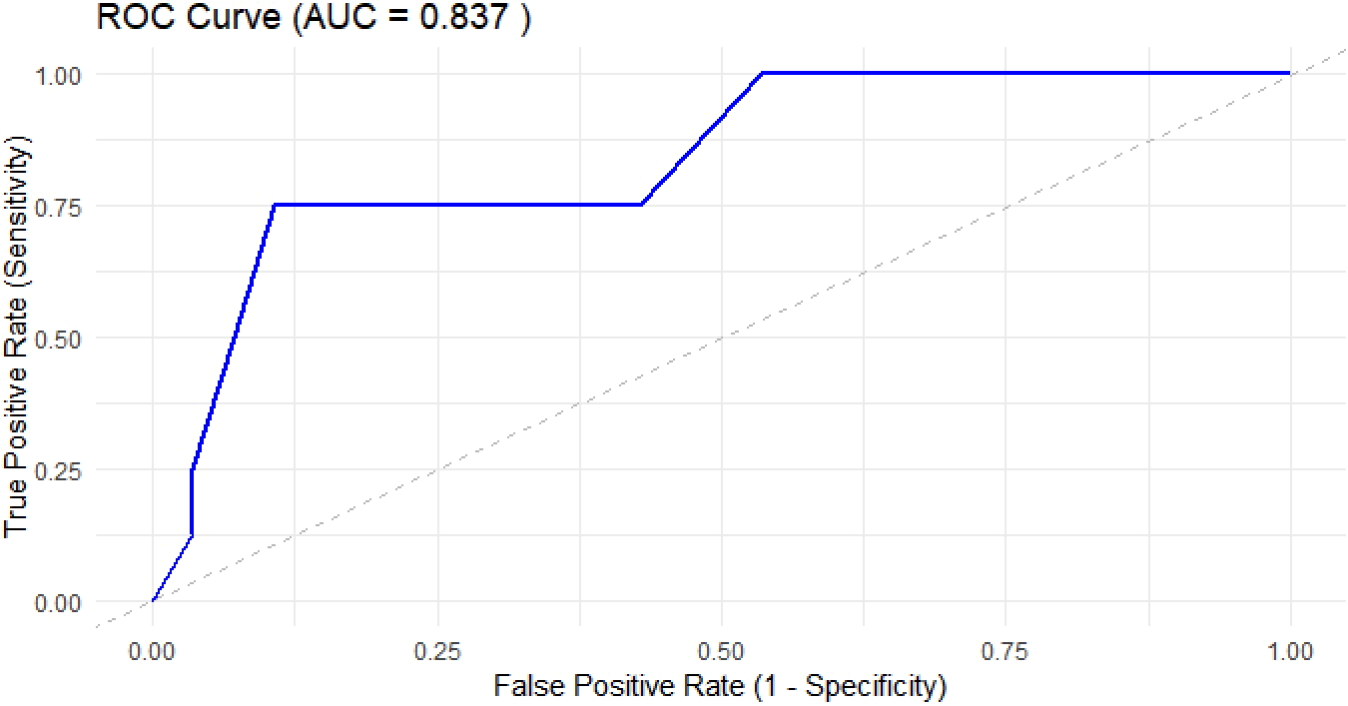
Receiver operating characteristic (ROC) curve for the IVIM-based model distinguishing myelofibrosis (MF) patients from non-MF MPN subjects and healthy controls. The area under the curve (AUC) is 0.837 (CI 95%: 0.71–0.97), indicating good diagnostic performance.

After combining the HCs group (Y = 0) and the non-MF subgroup (Y = 1), logistic regression again showed that higher f values significantly reduced the probability of MF (Y = 2). The regression coefficient for f was –19.2 (± 5.5), with a credible interval (95% CI: –30.3; –8.1), AUC = 0.837, confirming that reduced splenic perfusion is a differentiating factor for MF in the combined non-MF population. Although it is usually not recommended to combine healthy and sick patients into one comparative group, in this case it was justified due to the similarity of perfusion imaging between HCs and non-MF. The model converged well, and the number of effective samples was sufficient (Rˆ = 1), which indicates statistical reliability of the results (**Figure 3**).

This statistical procedure allowed for obtaining a greater “contrast” and increased the power of the test, constituting a relatively strong additional evidence for the predictive value of the f parameter, at least in the given observational setting.

Finally, spleen volume did not appear to have a meaningful effect on the probability of MF across the possible outcomes. Although differences in spleen volume were observed between groups, the regression estimates’ credible intervals included zero, indicating insufficient evidence to conclude that volume alone significantly alters MF risk. The regression coefficient for spleen volume in relation to the probability of outcome µ1 was –0.29 (95% CI: –2.91 to 2.31, estimated error 1.31), and for outcome µ2, 1.56, (95% CI -0.15-3.90, estimated error of 1.02). In both cases, the inclusion of zero within the credible intervals suggests that spleen volume is not a valid predictor of MF probability.

**Figure 4:**
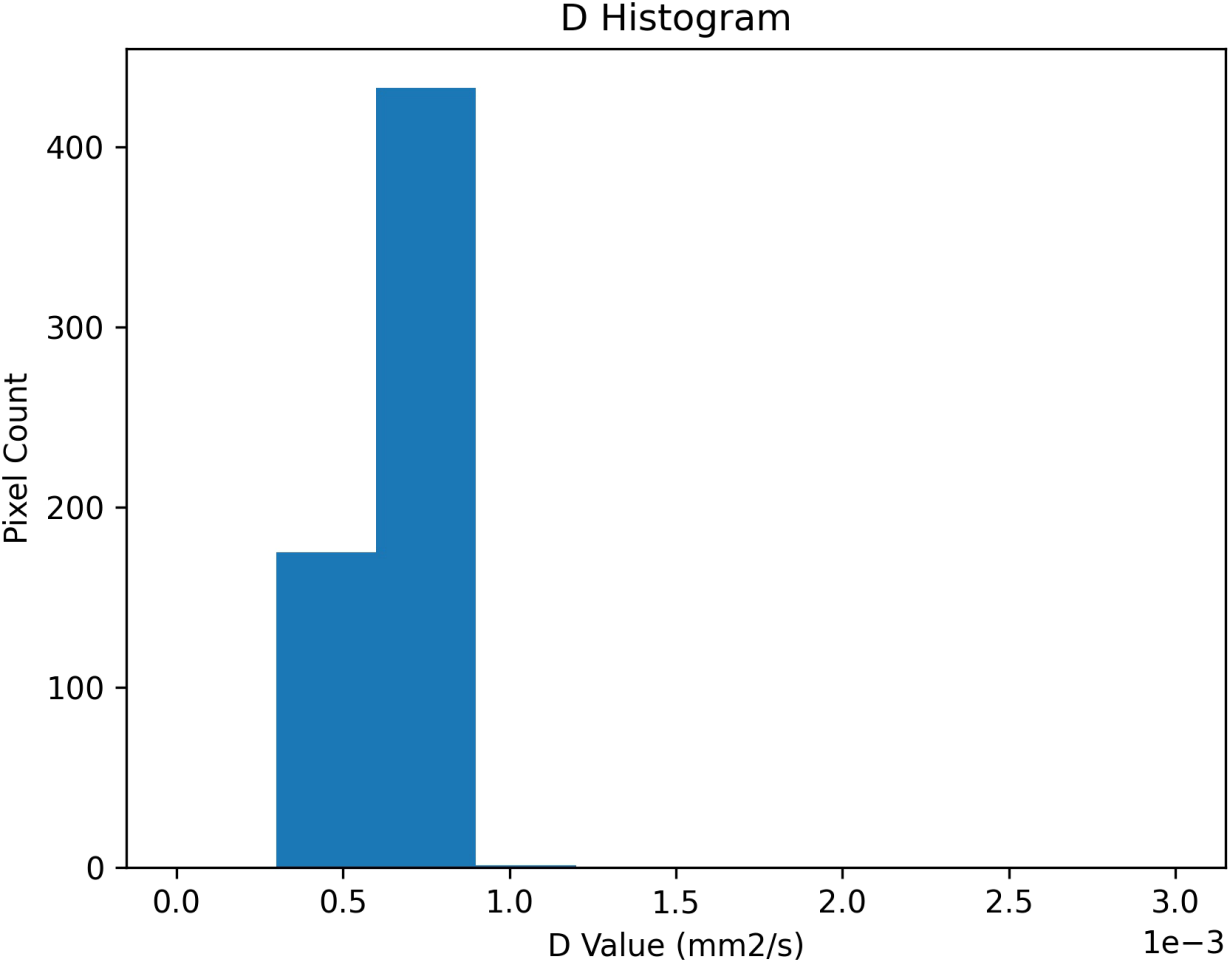
Histogram of pixel-wise diffusion coefficient (D) values [mm²/s] extracted from IVIM analysis of ROIs. Unimodal distribution centered around 0.7 × 10^-^³ mm²/s.

**Figure 5:**
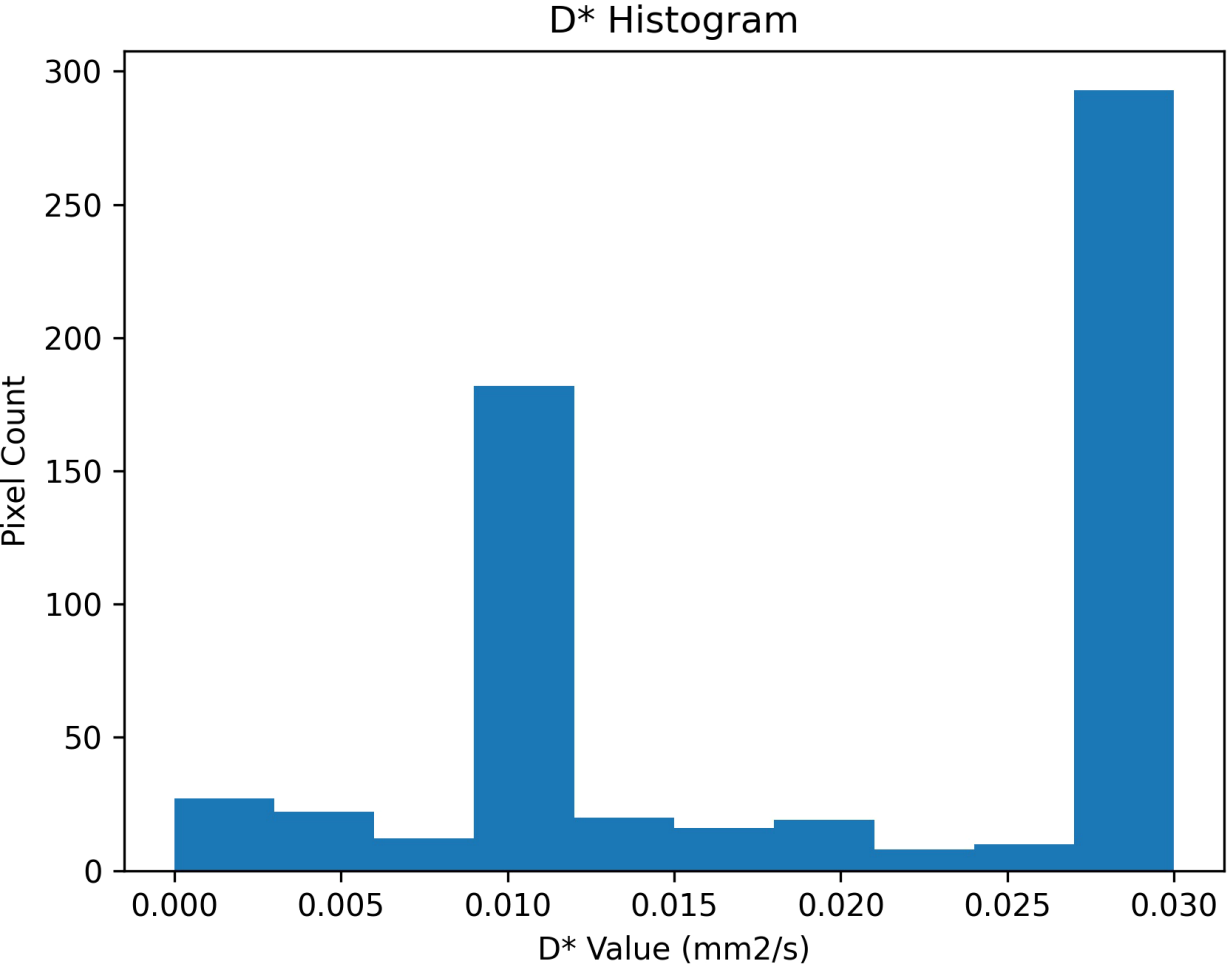
Histogram of pixel-wise pseudo-diffusion coefficient (D*) values [mm²/s] extracted from IVIM analysis of ROIs. Two dominant peaks near 0.01 and 0.03 mm²/s.

**Figure 6:**
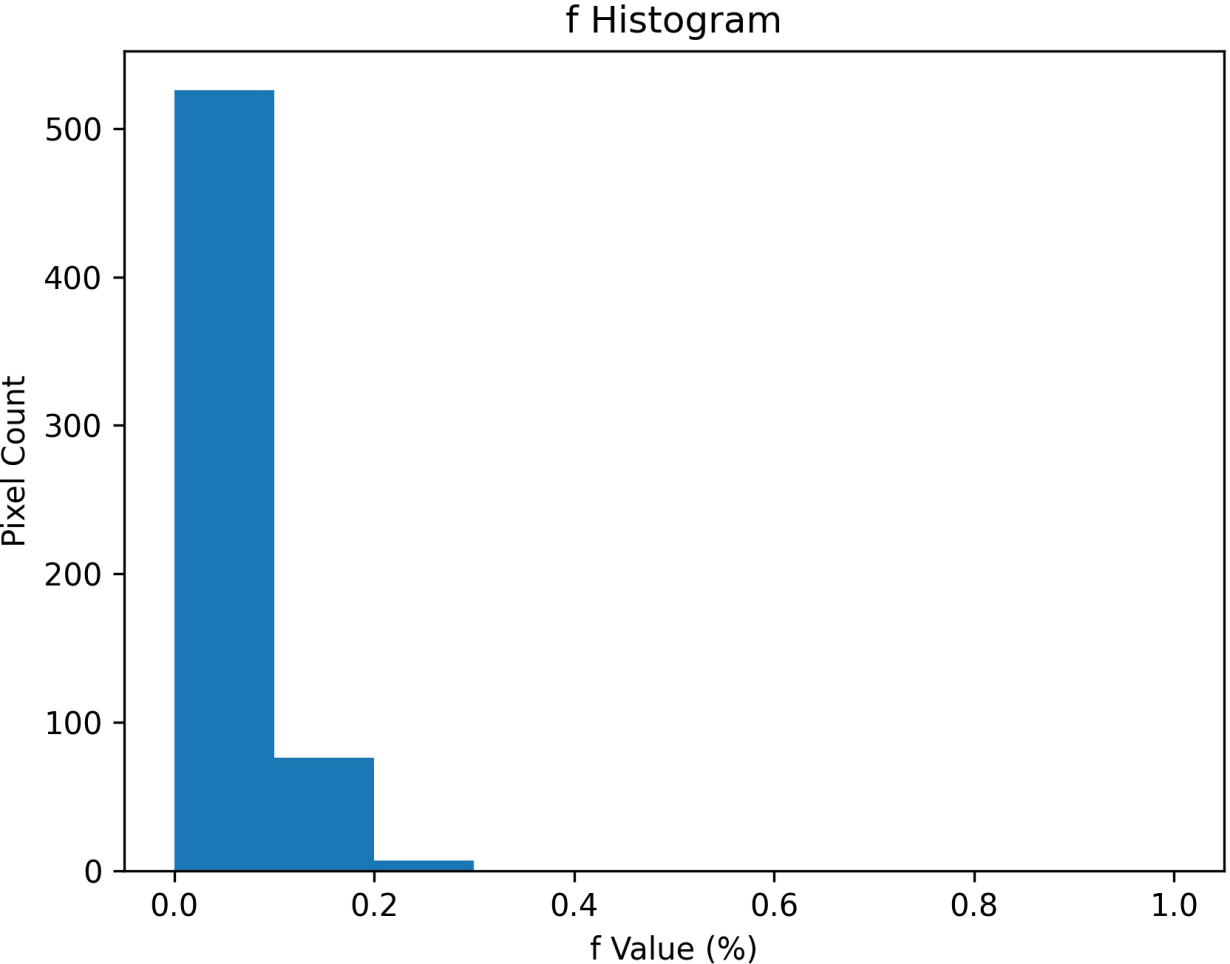
Histogram of pixel-wise perfusion fraction (f) values (%) extracted from IVIM analysis of ROIs. Left-skewed distribution, with most values falling below 0.2%.

To complement the central tendency measures reported above, a pixel-wise analysis of the distribution of IVIM parameters was performed within the ROIs segmented. Histograms for each parameter revealed distinct distributional features, with D (**Figure 4**) showing a unimodal and relatively narrow distribution, D* (**Figure 5**) displaying a bimodal pattern with clusters at 0.01 and 0.03 mm²/s, and f (**Figure 6**) showing so-called left-skew with values concentrated below 0.2%.

## 4 Discussion

Our study aimed to evaluate whether IVIM-derived parameters of the spleen— true diffusion coefficient (D) and perfusion fraction (f)—can serve as non-invasive biomarkers for early fibrotic transformation in the bone marrow among individuals with MPNs, in comparison with both healthy controls and patients diagnosed with MF.

Both D and f values were significantly lower in MF patients compared to controls and non-MF MPN individuals. This decline may be interpreted as a sign of reduced microvascular perfusion and restricted diffusion in the spleen, likely reflecting histopathological changes such as increased fibrosis, vascular remodeling, and altered cellularity (4, 26). As MF progresses, microcirculatory dynamics seems increasingly compromised within the spleen, and IVIM metrics become well suited to detect. However, it is important to emphasize that our study design was cross-sectional, and the reduced f values observed in MF patients can be interpreted as being associated with, [not yet predictive of, fibrotic transformation]. No histopathological validation of IVIM-derived f values was available in this desing.

From a physiological perspective, the f parameter used in this study reflects the splenic microcirculation and, in particular, may be more sensitive to fibrotic or hemodynamic changes characteristic of MF (1–4). The D parameter captures subtler changes in restricted diffusion related to cell density and interstitial changes, which may be less affected in the early stages of the disease (5–9).

The predictive value of f is supported by descriptive group differences, BFs, multinomial and binary logistic regressions with credible intervals, physiological modeling of spleen volume as a function of f, and [strong statistical convergence (Rˆ = 1)].

However, given the small number of MF cases (n = 8), the statistical power and precision of evidence required careful evaluation. Posterior comparisons of f values confirmed strong evidence value for differences between MF and HCs (BF = 18.4; 95% CI [0.012; 0.070]) and less stable results for MF versus non-MF patients (BF = 1.42; 95% CI [–0.006; 0.053]). Although the direction of effects remained consistent, the limited width and the overlap of credible intervals (including zero) indicate that estimates for intra-MPN comparisons should be interpreted with particular caution.

Some doubts at this stage may be raised by the predictive value of the D coefficient. In contrast to f, D shows only marginal group differences, a Bayes Factor near 1, and no significant predictive value in regression analyses, suggesting it is a less sensitive marker and less informative predictor for MF [in this setting].

Intergroup differences regarding to spleen volume are consistent with previous evidence linking splenomegaly to advanced disease stages (22, 24, 43, 44)—however BF regression confirmed that, because splenomegaly develops concurrently with MF, it reflects disease stage rather than offering a meaningful predictive value.

Our observations generally align well with earlier studies by Bian et al. (4) and their follow-up work (26), which identified D and f as strong indicators of splenic involvement and disease severity in hematologic malignancies, including acute leukemia. However, those studies used different patient cohorts and focused on AML/ALL rather than chronic MPNs. Therefore our findings extend the evidence base to MPN-associated MF, and demonstrate—within a histologically distinct and less aggressive disease group—that a reduction in IVIM parameters correlates with fibrotic transformation (**Table 5**). While both earlier and current studies employ IVIM methodology, our approach is distinguished by its direct comparison of MF and non-MF MPN patients, [which has not previously been comprehensively addressed in the existing literature].

**Table 5:** Regression models evaluating relationships between f and spleen volume.

Model	Intercept (Mean)	f coefficient (Mean)	V coefficient (Mean)	Residual SD (Sigma)
f ~ V [dm^3^]	-0.5 (± 0.1)	–	0.0 (± 0.2)	0.5 (± 0.1)
V[dm^3^] ~ f	0.0 (± 0.3)	-11.9 (± 4.4)	–	0.3 (± 0.0)

From a methodological standpoint, the use of IVIM in spleen imaging remains relatively novel (5, 35–39). Although previous studies have already confirmed the efficacy of IVIM in hepatic, pancreatic, and marrow applications (20, 25, 31, 45), this study makes a unique contribution by applying this technique to a population of patients with prefibrotic MPNs. [IVIM ability to detect microvascular abnormalities even before the appearance of overt myelofibrosis clearly indicates a potential role for D and f parameters as early predictors of subclinical progression]. Importantly, in contrast to spleen biopsy, IVIM imaging as a noninvasive technique, bypasses the complications and subjectivity associated with the diagnosis rendered on tissue material (46–49). Diagnostic interpretability also depends on the choice of analytic framework and validation metrics used (50, 51).

Our findings should be interpreted in light of several clinical variables that could influence splenic perfusion in patients (52–58). Although mutation status and treatment lines history (59) were partially available, the lack of full clinical annotation—particularly with regard to symptom burden, total duration of disease, or quantitative fibrosis scores—limits the body of evidence. Our study did not include histopathologic or molecular correlates of spleen perfusion. Consequently, our interpretation of reduced f values as reflecting fibrosis or microvascular remodeling requires farther research including validation against histologic standards (52–56).

The argument for considering the obtained results as scientific evidence (and therefore not spurious) is the [robustness of the model convergence (Rˆ = 1)] and the consistency of the analytical approaches used. At this stage, until further multicenter studies involving large patient groups are conducted, our results should therefore be interpreted as exploratory and hypothesis-generating.

Recent advances in non-invasive imaging techniques have opened new possibilities for tracking disease progression (60–63) in hematologic conditions, particularly chronic diseases. IVIM-based MRI, by simultaneously capturing microvascular perfusion and tissue diffusivity, offers a particularly promising approach. Our results support the hypothesis that spleen IVIM metrics, may be valuable as early biomarkers of fibrotic transformation (64–67).

Future studies should explore the potential of multi-organ IVIM assessment—particularly including the spleen, liver, and bone marrow—in a longitudinal approach. Simultaneous or sequential assessment of IVIM-derived perfusion and diffusion parameters in these organs may enable better characterization of the systemic manifestations of MPN, particularly the temporal dynamics of the underlying pathophysiological processes. The well-established role of extramedullary hematopoiesis and vascular remodeling in MF should be considered (54, 55). Current scientific knowledge on this topic, reinforced by our findings, indicates that assessing perfusion patterns in the spleen and liver, along with bone marrow changes, may help identify early subclinical changes (52, 60, 61). Longitudinal monitoring may be particularly important for further studies, as it could also provide information on the evolution of microcirculatory dysfunction over time and determine whether IVIM parameters respond to therapy or predict an unfavorable course (53, 63, 68) (acceleration) of the disease. A future integrated organ-level imaging strategy may ultimately facilitate personalized risk stratification and more precise therapeutic monitoring.

## Data Availability

The original contributions presented in the study are included in the article/supplementary material. Further inquiries can be directed to the corresponding author.
